# Monthly Summary

**Published:** 1878-01

**Authors:** 


### MONTHLY SUMMARY.

---

*A New Cause for Alarm.—Arsenic!*—A German Chemist has found arsenic in vulcanite supposed to be an impurity in the sulphur used for vulcanizing Caoutchouc. May not this unsuspected enemy be a cause of some of the sore mouths, the result of wearing these rubber plates. The Chemist rarely finds anything save the thing he is looking for, and as the efforts have all been in the direction of search after free mercury &c., would it not be well to investigate the Arsenic question ?

---

*Specific for Diptheria.*—Dr. Chapman, of Brooklyn, New York, claims Alcohol as a specific for diptheria, reducing the death rate from eighty-seven to the hundred cases to less than four. He combines with alcohol,(in the form of whiskey) quinine though the latter is not essential. He claims great success and says that he has never heard of but one drunkard having the disease, and states further that alcohol so administered has none of the intoxicating effects seen when given to persons in health. He considers alcohol as an antidote to the diptheritic poison.

---

*Phosphate of Lime in Relation to Fractures.*—In a recent thesis, M. Midrin, in reference to the question, whether the administration of phosphate of lime hastens the consolidation of fractures, comes to the following conclusions :—1. Phosphate of lime cannot be relied upon for this purpose. 2. Introduced directly into the economy, it is not absorbed when it is insoluble. 3. When given in a soluble form it is eliminated in the urine. 4. It is furnished in sufficient quantity by milk, bread, cereals, seeds, etc., and is assimilable only in this manner. 5. Its immediate action is only antacid and absorbent.—*Med. and Surgical Reporter.*

*Functions of the Liver.*—In a paper to the Paris Academy of Science, Dr. Claude Bernard contributed some new researches on the sugar-forming theory of the liver, and concluded :—

1st. That the glycogenic property is inherent in the tissue of the liver.

2nd. That this property manifests itself during life, and a certain time after death.

In the *Philadelphia Medical Times* of May 26th will be found an elaborate account by Dr. Lautenbach, of the results derivable from 283 experiments performed by him in Professor Schiff's laboratory, and leading to these conclusions:—1. The liver has for one of its functions the office of destroying certain of the organic poisons. 2. A poison is being constantly formed in the system of every animal, which it is the office of the liver to destroy.—*Med. and Surgical Reporter.*

---

*Preparation of Celluloid.*—This substance, much used in dentistry, is prepared as follows :—

Paper is treated by a continuous process with five parts of sulphuric acid and two of nitric acid, which convert it into a sort of gun cotton. The excess of acid is removed by pressure, followed up by washing with abundance of water. The paste, when thus washed, drained, and partially dried, is ground in a mill, mixed with camphor, ground again, strongly pressed. dried under a hydraulic press, between leaves of blotting paper, cut, bruised, laminated, and compressed again in a special apparatus suitably heated. It is said to be hard, tough, transparent, elastic, fusible, becoming plastic and malleable at 125°. It ignites with difficulty, is decomposed suddenly at 140° without inflammation, and gives rise to reddish flames. It is inodorous, and does not become electric on friction.—*Med. and Surgical Reporter.*

---

*Colored Borax Varnishes.*—It is well known that an aqueous solution of borax is able to dissolve shellac, forming a kind of varnish, to which any desired color can be imparted by mixing with pigments. Major Dr. Kahl, of Dresden, has communicated to the Dresden branch of the Saxon Society of Engineers the results of a large series of experiments made with these varnishes. He reports that they are very cheap, and dry very quickly, but they scale off from wood too easily. When this varnish is colored black with India ink and applied to paper, it possesses a fine gloss, but other colors, especially carmine, when mixed with this solution acquire an impure shade, nnd many pigments cement together in this solution, forming a hard and totally useless mass. The black shoe polish sold for ladies' boots is often made by adding some black pigments to this shellac solution. For bronze boots, rosanilin may be dissolved in any alcohol varnish.—*Scientific American.*

*Mastic for Fastening India Rubber on Metals.*—A mastic for fastening India rubber on metals may be obtained by steeping gum-lac, in the form of pulverized scales, in ten times its weight of concentrated ammonia. A transparent mass is thus formed, which, at the end of three or four weeks, becomes fluid without the use of warm water. This substance, applied on India rubber, becomes hard, and completely impervious to liquids and gases.—*Chemist and Druggist.*

---

*To take Rust out of Steel.*—Place the article in a bowl containing kerosene oil, or wrap the steel up in a soft cloth well saturated with kerosene; let it remain twenty-four hours, or longer; then scour the rusty spots with brickdust. If badly rusted, use salt wet with hot vinegar: after scouring, rinse every particle of brickdust or salt off with boiling water; dry thoroughly; then polish off with a clean flannel cloth and a little sweet oil.—*Scientific American.*

---

*The Dangers of Ether.*—It has always seemed to us the height of folly to declare there could be no danger in any anæsthetic. The lesson taught by a late death from nitrous oxide, has it is to be hoped, been well learned, and we shall in future hear less of the absolute safety of any agent capable of depriving a person of all sensation. Some cases in which ether has been followed by alarming symptoms have lately been recorded. They have been termed syncope, bnt the word is not appropriate, as the heart continued to beat after respiration ceased. This is what should have been anticipated. When death is produced by ether the animals's heart continues to beat long after the arrest of respiration. The pulse is quickened by ether and maintains its force through a long state of anæsthesia. In these facts lies the safety of ether. But it should never be forgotten that there is danger at a certain stage, and the danger is from the side of the respiration, which at length ceases. Stertorous breathing proceeds from paresis of the muscles of the palate, and should lead to the ether being suspended. So respiration growing more and more shallow and less frequent is a warning and should not be overlooked. It is very rare that the heart fails—perhaps never. Pallor is rare, too, and should excite attention if it occur. But, we repeat, the danger of ether is from the side of respiration, that of chloroform from the heart, and this fact goes far to explain their relative safety. In chloroform narcosis the danger is much more sudden. Ether gives warning.—*The Druggists' Circular.*

*Bichromate of Potash as an Antiseptic.*—M. Laujorrois lately presented a note to the French Academy, on the antiseptic properties of bichromate of potash. Experiments had shown him that the addition of one-hundredth part of bichromate in ordinary water prevents the putrefaction of all sorts of organic matter, such as meat, urine, etc. A thousandth part of bichromate prevents beer from turning sour. After three months' immersion in a solution, meat was hardened and dry.—*Phar.*

---

*Properties of the Human Gastric Juice.*—M. Charles Richet has been studying these matters upon the person of the patient on whom Verneuil successfully performed gastrotomy. He has reached the following conclusions: 1. The acidity of the gastric juice, whether pure or mixed with food, is equivalent to 1.7 grammes of hydrochloric acid to a thousand grammes of fluid. 2. Acidity increases slightly at the end of digestion, and is independent of the quantity of liquid contained in the stomach. Wine and alcohol increase, but cane-sugar diminishes it. 3. If acid or alkaline matters are introduced, the gastric juice tends to return to its normal acidity. 4. The mean duration of digestion is from three to four and a half hours or more. Food does not pass successively, but in masses. 5. According to four analyses made by a modification of Schmidt's method, it was proved that free hydrochloric acid exists in the gastric juice. 6. It is possible to extract all the lactic acid contained in the stomach, and to prove that there is one part lactic acid to nine parts hydrochloric acid. 7. Following the method of Berthelot, that is, by agitation with anhydrous ether and deprived of alcohol, it can be shown that lactic acid is free in the gastric juice. 8 The question so long in controversy as to the nature of the free acid in the stomach seems almost solved, and it may be said that in every 1.000 grammes of gastric juice there are 1.53 grammes of hydrochloric acid and 0.43 of lactic acid.—*Lyon Medical.*

---

*Salicylic Acid and Borax.*—It may be interesting and perhaps useful for some readers of the *Journal* to know, that while a solution containing ten grains of salicylic acid and ten grains of borax in one ounce of water, has a very bitter taste and an acid reaction, a solution containing ten grains of salicylic acid and fifteen grains of borax has no disagreeable taste, and is nearly neutral. This solution appears to possess all the valuable properties of salicylic acid, and forms an agreeable means of using the acid internally or as a gargle.—S. N.—*Lond. Pharm. Journal.*

*Nitrite of Amyl.*—Mr. W. Lemon Lane, M. B., as the result of experiments upon kittens, publishes the following conclusions:

1. Amyl nitrite, when inhaled in small quantities, produces reddening of the face in man, and of the nose and mouth in kittens; this action is due, according to Brunton, to the dilatation and overfilling of the arterioles.

2. Large quantities, by inhalation, produce in kittens cyanosis of the nose and the mouth, along with insensibility. The former arises from over-distension of the venous system, this being due to the engorged arterioles propelling the blood into the veins, while the insensibility probably arises from over-distension of the venous system and of the heart.

3. When inhaled in small quantities it produces recovery from chloroformic insensibility by dilating the arterioles of the brain, and thus removing the cerebral anæmia due to the chloroform.

4. When inhaled in large quantities, instead of producing recovery from chloroformic insensibility, it not only retards it but it may cause death by paralysis and over-distension of the heart and engorgement of the venous system.

5. It causes a rise of temperature when inhaled in small quantities, by the increased amount of blood in the arterioles causing an increased tissue change in the body.

6. In large doses (inhaled) it produces a fall of temperature.

7. It also helps to produce recovery from the chloroform insensibility by raising the temperature, which is always lowered by chloroform, and by removing the paralysis of the heart due to chloroform; this action is well seen by the nitrite of amyl making the heart's beat fewer and its sounds louder.

8. Death is caused chiefly by paralysis of the heart, which is shown by all its cavities being distended, and by engorgement of the venous system.—*British Med. Journal.*

---

*Atheroma.*—At the sitting of the Society of Public Medicine (Paris, June 27th,) M. Gubler communicated an interesting paper on arterial atheroma. He believes many influences are at work in producing this alteration; whilst the rich in their old age are often exempt from it, the role of alcohol, as a cause, has been blamed too much; the rich drink as much as the poor; alimentation plays a more important part: the wealthy have a more succulent and azotized diet, whilst the workman eats such vegetables as horse-radish and apples, which contain a large quantity of calcareous salts. The frequency of atheroma among those who abuse vegetable diet is due to these calcareous salts, in virtue of general laws. The algæ retain the iodine and bromine they take up in the sea, and from analogy, he considers that mineral solutions leave their salts in the tissues. Cretification especially takes place when the tissues are not well nourished ; the middle coat of the arteries belongs to this class, and is supported by imbibition. M. Gubler thinks it worthy of research to compare the frequency of atheroma among the rich who adopt a vegetable diet. The rarity of atheroma in localities poor in silica has been established, whilst Dr. Leblane has verified its presence in Orleans, a lime formation, and Dr. F. Raymond has noticed indurated arteries in many young monks of the order of Chartreux, who lived exclusively on legumes.—*Med. and Surg. Reporter.*

---

*A Daring Therapeutist.*—At a late meeting of the Massachusetts Dental Society, Dr. Waters, of Salem, stated that bicarbonate of soda, such as used for cooking purposes, or any other alkali in neutral form, would afford instantaneous cessation of pain from the severest burns or scalds, and would cure such injuries in a few hours. Dipping a sponge into boiling water, the Doctor squeezed it over his right wrist, producing a severe scald around his arm and some two inches in width. Then, despite the suffering occasioned, he applied the scalding water to his wrist for half a minute. Bicarbonate of soda was at once dusted over the surface, a wet cloth applied, and the pain the experimenter stated, was almost instantly deadened. Although the wound was of a nature to be open and painful for a considerable time, on the day following the single application of the soda the less injured portion was practically healed, only a slight discoloration of the flesh being perceptible. The severer wound, in a few days, with no other treatment than a wet cloth kept over it, showed every sign of rapid healing.—*Medical and Surgical Reporter.*

---

*The Scientific American* is the most popular scientific paper published, and has now reached its *thirty-third* year. It is splendidly illustrated, and is a useful paper, for the professional man as well as for the mechanic, affording to every intelligent mind a constant supply of instructive reading. Terms $3.20 including postage. Published weekly by Munn & Co., 37 Park Row, New York City.

---

*The American Agriculturist.* This is also one of the most useful and entertaining periodicals published, and will well repay the cost of subscription, which is the small sum of $1.50 per annum, with ten cents for postage. The monthly numbers are beautifully illustrated, and the reading matter is both interesting and instructive to all classes. Publishers, Orange, Judd & Co., 245 Broadway, New York City.

